# The prevalence of hand and wrist osteoarthritis in elite former cricket and rugby union players

**DOI:** 10.1016/j.jsams.2019.03.004

**Published:** 2019-08

**Authors:** Mary E. Jones, Madeleine A.M. Davies, Karishma Shah, Simon Kemp, Nick Peirce, Kirsten M. Leyland, Keith A. Stokes, Andrew D. Judge, Julia L. Newton, Dominic Furniss, Nigel K. Arden

**Affiliations:** a Nuffield Department of Orthopaedics, Rheumatology, and Musculoskeletal Sciences, University of Oxford, Botnar Research Centre, UK; b Rugby Football Union, UK; c England and Wales Cricket Board, National Cricket Performance Centre, UK; d Department of Primary Care and Population Health, University College London, UK; e Department of Health, University of Bath, UK; f Bristol Medical School, Population Health Sciences, University of Bristol, UK; g Bristol Medical School, Translational Health Sciences, University of Bristol, UK

**Keywords:** Epidemiology, Athlete, Injury, Prevalence, Aging

## Abstract

**Objectives:**

This study aimed to determine the prevalence of hand and wrist osteoarthritis in former elite cricket and rugby union players, by sport and playing position, and to define the prevalence of severe hand injury, and its association with hand osteoarthritis.

**Design:**

Cross-sectional.

**Methods:**

Data from cross-sectional studies of former elite male cricket and rugby players were used to determine the prevalence of hand pain, physician-diagnosed osteoarthritis, and previous severe injury. Multivariable logistic regression was used to determine the association of previous injury with pain and osteoarthritis.

**Results:**

Data from 200 cricketers and 229 rugby players were available. Complete case analysis resulted in 127 cricketers and 140 rugby players. Hand pain was more prevalent amongst cricketers (19.7%) than rugby players (10.0%). The prevalence did not differ between cricket and rugby players for hand osteoarthritis (2.4% and 3.6%), wrist osteoarthritis (1.6% and 2.1%), or previous severe hand injury (36.2% and 31.4%). No significant association between previous hand injury and pain or osteoarthritis was identified in either sport.

**Conclusions:**

Former elite cricketers reported more hand pain than rugby players. No significant association was found between self-reported severe injury and hand osteoarthritis in either cohort, potentially indicating that risk factors aside from injury may be more prominent in the development of hand osteoarthritis.

## Practical implications

● No association was found between previous severe hand injury and hand pain or osteoarthritis in either former elite cricket or rugby players.

● The relationship between injury and osteoarthritis may not be as readily defined for the hand and wrist as for the hip and knee.

● Factors other than injury, such as micro-trauma and cumulative loading, may be more prominent in the development of hand and wrist osteoarthritis.

## 1 Introduction

Osteoarthritis (OA) is a substantial health care burden worldwide. OA is associated with chronic pain and loss of function, which makes it a leading cause of disability in adults.^1^ OA of the hand and wrist can develop relatively early in life, and therefore has the potential to substantially affect individual and societal productivity.^2^ In the general population, symptomatic, radiographic hand OA in men has been estimated at 8.2%, while the prevalence of hand or wrist pain during 3 of the last 12 months has been estimated to affect 9.5% of men.^3^, ^4^ Hand OA commonly presents as finger pain and stiffness in the interphalangeal joints (IPJs), and Heberden and Bouchard’s nodes. It also commonly affects the first carpometacarpal joint (CMCJ), which may result in pain that radiates to the wrist, decreased grip strength, and a subluxed joint.^5^ Wrist OA often affects the radio-carpal, radio-ulnar, and mid-carpal joints.

The aetiology of hand and wrist OA is multifactorial. Previous research has identified risk factors including genetic predisposition, handedness, and repetitive hand use, whilst the relationship with other risk factors such as obesity, smoking and bone mineralization is still not yet well established.^6^, ^7^ The potential for repetitive fine movement and joint loading to contribute to the development of OA has encouraged the consideration of physical activity and sports participation as risk factors for the development of OA. However, the relationship between physical activity, sports participation and OA remains poorly defined, particularly for joints other than the hip and knee. Studies of elite rock climbers have suggested an increased risk of radiographic hand OA,^8^ while moderate sporting activity has been shown to be protective against lower limb OA, through joint loading and cartilage synthesis.^9^, ^10^ Study of the association between injury and OA in former elite sporting populations has been largely limited to the knee and hip.^11^, ^12^, ^14^

In the general population, half of acute sporting injuries requiring continued management after presentation at an Orthopaedic department are to the hand or wrist, and of the sporting activities recorded for these injuries, rugby and football were the most common.^16^ Non-collision sports, such as cricket, have a lower overall injury rate than contact and collision sports, with hand injury incidence estimated at 0.1 per 100 player-days during the ICC Cricket World Cup 2011.^17^ Despite the popularity of rugby and cricket, there has been limited study of longer-term musculoskeletal health at the amateur and professional levels of these sports. Research into the epidemiology of hand OA has been limited, with a substantial focus on lower limb OA. As part of an Arthritis Research UK Centre for Sport, Exercise and OA initiative to better define the relationship between sport, exercise and OA, we aimed to describe the prevalence of hand pain, hand OA, and wrist OA in former elite cricket and rugby union players, and to determine whether there was an association between injury history and reported hand pain or OA.

## 2 Methods

Cross-sectional questionnaire studies of former players were undertaken within the Arthritis Research UK Centre for Sport, Exercise and Osteoarthritis. Epidemiological questionnaires were developed to collect lifetime injury, medical and playing histories for former elite athletes in specific sports, and underwent player involvement groups, similar to public and participant involvement (PPI) in healthcare research.^18^, ^19^, ^21^ Data from studies of former elite cricket and rugby union players were used due to the specific repetitive movement patterns at the hand and wrist within cricket and rugby, such as batting, bowling, grappling, passing and catching, and the prominence of hand and finger injuries in these sports.

For both the cricket and rugby studies, ethical approval was received (NHS REC 15/LO/1274 and University of Oxford MSD-IDREC-C1-2014-020 respectively), informed consent was obtained from all participants or their next of kin where appropriate, and the rights of participants were protected. Former players were invited to participate in the questionnaire studies by the Professional Cricketers’ Association, and by membership organisations of elite rugby union players (the England Rugby Internationals Club, and the University of Oxford and University of Cambridge Rugby Football Clubs). Participants in both studies could complete the questionnaire online, by telephone, or through a postal questionnaire, reminder communications were sent, and participant consent and study data were managed using REDCap electronic data capture software hosted at the University of Oxford.^22^

Self-reported hand pain prevalence was collected using the National Health and Nutrition Examination Survey (NHANES) criteria, which asked whether a participant experienced pain in their hand on most days of the last month. NHANES pain can indicate symptomatic or early, undiagnosed OA. OA prevalence was determined using self-reported physician-diagnosed OA for the hand and wrist. The demographics of the playing cohorts were collected (age, weight, smoking status, ethnicity, predominant playing position, years since retirement).^18^, ^19^ Playing position was defined in cricket as wicketkeeper, batsmen or bowlers and all-rounders; and in rugby as forwards or backs. Severe hand injury was defined as a self-reported injury resulting in more than 4 weeks of reduced participation in sport, exercise or training.

Players without complete data for subject characteristics, sport-specific variables, and outcome measures were excluded due to missing data; those reporting rheumatoid arthritis, or still playing elite rugby or cricket were also excluded (Supplementary Fig. 1).

Two-sample t-tests were used to compare the demographics between cohorts. The prevalence of NHANES pain and OA at the hand, and OA at the wrist was estimated for the cricket and rugby samples, and by playing position. Chi-squared tests were used to compare the prevalence of each outcome between the sport samples. Univariable logistic regression was used to determine the association of side-specific (left or right) previous severe hand injury with hand pain and OA in each cohort. Multivariable models were adjusted for age, height and weight. Body mass index is a potentially limited metric in athletic populations and was not used.^23^, ^24^

## 3 Results

Data for 200 cricket and 229 rugby union players were available for analysis. Of these participants, 127 cricketers and 140 rugby players were included in complete case analysis (Supplementary Fig. 1). Differences between excluded and complete case participants were seen in weight and years since retirement, with excluded rugby and cricket players being significantly heavier, and excluded cricket players having a longer time since retirement (Table 1 and Supplementary Table S1).

**Table 1:** Participant characteristics for former cricket and rugby players included in analysis.

	Complete case cricket sample (n = 127)	Complete case rugby sample (n = 140)
Age (years)		
Mean (SD)	56.4 (14.0)^a^	60.4 (16.0)^a^
Range	28–84	28–95
Height (m)		
Mean (SD)	1.8 (0.1)	1.8 (0.1)
Range	1.7–2.0	1.7–2.0
Weight (kg)		
Mean (SD)	88.5 (11.6)^a^, ^b^	92.1 (13.5)^a^, ^b^
Range	63.5–123	63.5–130.2
Smoking status [N (%)]		
Current smoker	5 (4%)	4 (2.9%)
Does not smoke	109 (85.8%)	130 (92.9%)
Ex-smoker	13 (10.2%)	6 (4.3%)
Ethnicity [N (%)]		
White	121 (95.3%)	136 (97.1%)
Non-white	6 (4.7%)	4 (2.9%)
Handedness [N (%)]		
Right	102 (80.3%)	117 (83.6%)
Left	10 (7.9%)	15 (10.7%)
Both	15 (11.8%)	8 (5.7%)
Playing position [N (%)]		
Wicketkeepers	17 (13.4%)	–
Batsmen	35 (27.6%)	–
Bowlers/all-rounders	75 (59.0%)	–
Forwards	–	63 (45.0%)
Backs	–	77 (55.0%)
Time since retirement (years)		
Mean (SD)	23.7 (13.7)^a^, ^b^	27.7 (16.0)^a^
Range	2–57	1–63
Previous severe hand injury [N (%)]	46 (36.2%)	44 (31.4%)

a Denotes differences in characteristic between cricket and rugby samples (p < 0.05).

b Denotes differences in characteristic between each sport’s complete case and excluded samples (p < 0.05).

Of those included in complete case analyses, former rugby players included were significantly older, heavier, and had been retired from elite sport longer than included former cricketers (Table 1). The majority of cricket players were bowlers/all-rounders, and the majority of rugby players were backs (Table 1). Both groups reported similar prevalences of severe hand injury (36.2% of cricket players and 31.4% of rugby players).

Hand pain was significantly more prevalent in former cricket players than former rugby players (19.7% versus 10.0%, p = 0.025; Table 2). Wicketkeepers reported the most hand pain (35%) but no hand or wrist OA (Table 2). Among rugby players, forwards reported more hand pain (14.3%) and hand OA (4.8%) than backs. Wrist OA was relatively uncommon in both sport samples, but reported more for bowlers (2.7%) and backs (2.6%; Table 2).

**Table 2:** The prevalence and 95% confidence interval (CI) of previous severe hand injury, hand pain, hand OA and wrist OA in former cricket and rugby player samples and by playing position.

Cricket sample								
	All players (N = 127)		Wicketkeepers (N = 17)		Batsmen (N = 35)		Bowlers/all-rounders (N = 75)	
	N	% (95% CI)	N	% (95% CI)	N	% (95% CI)	N	% (95% CI)
Severe hand injury	46	36.2% (28.2–45.0%)	5	29.4% (11.5–57.1%)	14	40.0% (24.7–57.6%)	27	36.0% (25.8–47.7%)
NHANES hand pain	25	19.7% (13.6–27.6%)^a^	6	35.3% (15.2–62.3%)	6	17.1% (7.6–34.3%)	13	17.3% (10.2–27.9%)
Physician-diagnosed hand OA	3	2.4% (0.8–7.2%)	0	–	1	2.9% (0.4–19.2%)	2	2.7% (0.6–10.3%)
Physician-diagnosed wrist OA	2	1.6% (0.4–6.2%)	0	–	0	–	2	2.7% (0.6–10.3%)

Rugby sample						
	All players (N = 140)		Forwards (N = 63)		Backs (N = 77)	
	N	% (95% CI)	N	% (95% CI)	N	% (95% CI)
Previous severe hand Injury	44	31.4% (24.2–39.7%)	25	39.7% (28.1–52.5%)	19	24.7% (16.2–35.8%)
NHANES hand pain	14	10.0% (6.0–16.3%)^a^	9	14.3% (7.5–25.6%)	5	6.5% (2.7– 14.9%)
Physician-diagnosed Hand OA	5	3.6% (1.5– 8.4%)	3	4.8% (1.5–14.1%)	2	2.6% (0.6–10.1%)
Physician-diagnosed Wrist OA	3	2.1% (0.7–6.5%)	1	1.6% (0.2–10.9%)	2	2.6% (0.6– 10.1%)

a Denotes differences in characteristic between cricket and rugby samples (p < 0.05).

No significant relationship was found between previous hand injury and hand pain or OA in either former cricket or rugby players. This result was maintained in adjusted multivariable regression analyses (Table 3).

**Table 3:** The association between previous hand injury, and hand pain and hand OA, in former cricket and rugby players.

	Hand pain		Hand OA	
	Unadjusted odds ratio (95% CI)	Adjusted^a^ odds ratio (95% CI)	Unadjusted odds ratio (95% CI)	Adjusted^a^ odds ratio (95% CI)
Cricket hand injury				
Left	1.23 (0.37–4.14)	1.24 (0.35–4.38)	–	–
Right	1.38 (0.50–3.80)	1.51 (0.53–4.27)	1.22 (0.11– 13.90)	1.43 (0.12–17.03)
Rugby hand injury				
Left	3.05 (0.56–16.70)	3.40 (0.56–20.7)	4.39 (0.37–51.59)	2.87 (0.22–38.15)
Right	1.19 (0.30–4.70)	1.03 (0.25–4.3)	1.18 (0.12–11.74)	1.07 (0.10–11.44)

a Analyses adjusted for age, weight and height.

## 4 Discussion

Hand pain was significantly more common in former elite cricketers (19.7%) than rugby union players (10.0%). The overall prevalence of previous severe hand injury was similar in both groups, with 36.2% of former cricket players and 31.4% former rugby players reporting a severe hand injury. There was no statistically significant association between side-specific hand injury and pain or OA in cricket or rugby players.

Strengths of this study include the analysis of hand and wrist OA in the largest cohorts of former elite cricket and rugby players collected for the study of joint pain and OA. Physician-diagnosed OA and NHANES pain are specific OA outcome measures frequently used for lower limb OA, which capture both patient and health-service relevant OA-related outcomes. This study also benefits from parallel methodology in cricket and rugby, which has resulted in, to the authors’ knowledge, the first direct inter-sport comparison of long-term musculoskeletal health between elite sports cohorts, which has the capacity to help inform risk management in sport.

Limitations of our study include the relative rarity of hand and wrist OA, which has resulted in comparatively few cases, particularly where subgroups such as playing positions are presented. A greater sample size may have improved our power to detect any association between injury and pain or OA, and would have increased the reliability of our findings. Secondly, whilst common in the epidemiology of OA, self-reported outcomes may not be precise measurements of OA, and may not be reflective of radiographic, early, or subclinical OA. For hand OA clinical trials, American College of Rheumatology (ACR) criteria have been recommended.^25^ However, ACR criteria include features of hard tissue enlargement and deformity, which may not be indicative of the clinical or symptomatic burden of hand OA in sporting populations with a history of fracture, and additionally would not be feasible to reliably measure in a cross-sectional questionnaire study.

Compared with radiographic or subclinical OA, outcome measurements of self-reported physician-diagnosed OA may have underestimated the prevalence of OA in this study. A potential underestimation of OA cases may have reduced our power in analyses, particularly subgroup analyses, and also decreased our likelihood of detecting an association between groups with and without OA following injury. NHANES pain was used in addition to physician-diagnosed OA to measure potential early or subclinical OA. Furthermore, former elite athletes may have differences in pain threshold, coping, healthcare provision and clinician access. These factors may not be different between these sporting populations, but will affect the validity of any comparison with the general population.

Base of thumb OA (first CMCJ) often presents as hand or wrist pain, and distinguishing between pain radiating from the wrist, CMC or IP joints may not be straightforward.^5^ This study did not differentiate between finger, thumb, hand and wrist pain. Therefore, it is possible that some participants may have considered first CMCJ OA as wrist pain, which was not recorded for both cohorts, and resulted in an underestimate of hand pain and hand OA prevalence. Future studies may benefit from visual aids in order to more accurately distinguish between hand, wrist, IP and first CMCJ pain and OA. Validation of this reporting with clinical records and diagnoses would clarify the accuracy of self-reported hand OA.

The timeframe of the severe injury definition was used to reduce recall bias. However, it may underestimate the total burden of hand injury, by excluding injuries resulting in less than four weeks of reduced participation in exercise and sport. Potentially underestimating hand injury in this way may decrease the likelihood of identifying an association between injury and hand pain and OA. Finger injuries, such as dislocations, may be managed pitch-side or non-surgically, enabling continued participation despite injury. Previous research in community rugby has identified that whilst there may be on-pitch medical attention for hand injuries every 3 games (14 injuries per 1000 player hours), only 10% of these hand injuries will result in removal from play.^26^ Similar situations may arise in cricket, though the type of injury and fine motor skills required of the player may prevent return to play as readily as in rugby. Injuries requiring on-pitch medical attention but not resulting in timeloss from sport may not be captured in this study. Equally, injuries that met this study’s injury definition of required timeloss from sport may have benefitted from sufficient recovery, supporting an ultimately better outcome than for those returned to play or not removed. Therefore, repeated minor injuries or earlier return to play may have contributed negatively to the hand and wrist outcomes in these players.

A final potential limitation of this study is the reported sample only includeing male former elite athletes. Whilst this population may comprise the former athletes with the highest injury rates^16^, ^27^ and be reflective of the sex forming the majority of elite English rugby and cricket players, women are at a higher innate risk of hand OA due to the likely effect of oestrogen and other hormonal risk factors on the development of hand OA.^28^ Further research amongst female athletes would help to clarify these risk factors for hand OA and for female athlete musculoskeletal health. Consideration of other risk factors for hand OA, such as occupation after retirement from sport and family history of OA, may improve precision of results in future work.

The aims of this study were to describe the prevalence of hand pain, hand OA, and wrist OA, and determine the association of injury history with hand pain and OA in former elite cricket and rugby players. Hand pain was found to be higher in cricket players than rugby players, whereas the prevalence of hand and wrist OA did not differ between cricket and rugby players. The difference between sports for pain but not OA may be due to under-diagnosed OA in the cricket players or under-reporting of pain in the rugby players. The prevalence of hand pain reported by rugby players was similar to that of a Dutch general population study, which found 9.5% of men with hand or wrist pain during 3 of the last 12 months.^3^ The hand pain prevalence reported by cricketers was approximately double that of the Dutch men. However, the study of Dutch men reported hand and wrist pain as one outcome and used a different pain definition, which limits comparability with our findings. The prevalence of symptomatic, radiographic hand OA in men from the general population has been estimated at 8.2%, higher than the OA prevalence reported by these cricket and rugby players.^4^ However, radiographic OA is expected to be more prevalent than symptomatic OA.

Whilst the association between injury and post-traumatic OA has been well described in the lower limb, our study found that the lifetime prevalence of severe hand injury did not differ between former elite rugby and cricket players, and that there was no association of injury with the OA-outcome measurements of NHANES pain or physician-diagnosed OA. Given the association of injury with OA in the lower limb, the reported prominence of avulsion and digit fractures in rugby,^29^and the hand and wrist injuries in cricket,^17^ the lack of association between injury and OA and pain was unexpected. However, given the complex anatomy of the hand and wrist, the relationship between injury and OA may not be as readily defined for the hand and wrist as for other joints. Further work is needed to clarify whether our negative finding may be resultant of being underpowered for our analysis, the injury definition used, or the lack of accuracy in injury and pain reporting. However, the lack of association may also be indicative of risk factors other than injury being more important in the development of hand and wrist OA.

High impact physical activity and repetitive hand movements have been described as risk factors for the development of hand OA.^6^ It is possible that repetitive non-injurious movement, or cumulative micro-trauma may have contributed to hand and wrist OA in these former elite athletes. Over their sporting careers, these athletes may have sustained chronic joint loading and repeated impact from catching or being struck by the ball in cricket, and from grappling, lifting and gripping in the tackle in rugby. Prolonged exposure to repeated impact and micro-trauma may have contributed to development of hand pain and OA in these populations. Further work should seek to define measures of loading or micro-trauma at the hand and wrist. Radiographs should be used to inform whether these factors may be occupational risks for structural musculoskeletal change and ultimately hand and wrist OA, as hand pain reported in this study may be undiagnosed hand OA.

A systematic review of risk factors for the development of hand OA found age to be the most influential risk factor and also suggested a genetic predisposition is central to the development of hand OA.^6^ We have not sought to identify genetic components for hand OA in our sporting populations, though these may have contributed to pain and diagnoses. Increasingly there is understood to be a metabolic component to OA, and in a cohort of middle-aged women, metabolic syndrome has been shown to be associated with the development of painful IPJ hand OA.^30^ There were significant differences in weight between our cricket and rugby samples, and the complete case analysis excluded players who were heavier in both sporting populations. However, as pain was more prevalent in cricketers, who had a significantly lower weight than rugby players, it is possible that the metabolic component of OA is less influential in hand OA pathogenesis in these male sporting professionals compared to other non-sporting populations.

The generalisability of this study is limited to the elite sporting populations and potentially to broader cricket and rugby-playing populations, though these are two sports with very high participation rates.

## 5 Conclusions

This study has found the prevalence of hand pain to be significantly higher in former elite cricket players compared to rugby union players (p = 0.025). Meanwhile, the prevalence of hand and wrist OA were comparable in both groups. Unexpectedly, we found no evidence of an association between sporting hand injuries, and hand OA in either cohort. While hand injuries may have been underestimated due to the severe injury definition, this study may suggest that risk factors aside from injury, such as chronic load or repetitive micro-trauma, may be more prominent in the development and progression of hand and wrist OA in former elite male athletes.
