# Supplementary material for: The prevalence of hand and wrist osteoarthritis in elite former cricket and rugby union players
Source: J Sci Med Sport. 2019 Aug;22(8):871–5. doi: 10.1016/j.jsams.2019.03.004 (PMC6593259; doi:10.1016/j.jsams.2019.03.004)
Supplement: Supplementary file 1 [file mmc1.docx]

**Supplementary Material**

**Table A.1.** Differences between participant characteristics for former cricket and rugby players included in analysis, and those excluded for the use of complete case analysis.

|  | Complete Case Cricket Sample (n=127) | Excluded Cricket Sample (n=66) | Complete Case Rugby Sample (n=140) | Excluded Rugby Sample (n=89) |
| --- | --- | --- | --- | --- |
| Age (years) |  |  |  |  |
| Mean (SD) | 56.4 (14.0)* | 57.8 (15.2) | 60.4 (16.0)* | 62.4 (16.0) |
| Range | 28 - 84 | 29 - 88 | 28 - 95 | 22 - 86 |
| Height (m) |  |  |  |  |
| Mean (SD) | 1.8 (0.1) | 1.8 (0.7) | 1.8 (0.1) | 1.8 (0.1) |
| Range | 1.7 - 2.0 | 1.65 - 1.95 | 1.7 - 2.0 | 1.7 - 2.0 |
| Weight (kg) |  |  |  |  |
| Mean (SD) | 88.5 (11.6)*† | 92.5 (16.7)† | 92.1 (13.5)*† | 97.4 (17.8)† |
| Range | 63.5 - 123 | 71.7 - 200 | 63.5 - 130.2 | 66.2 - 180 |
| Smoking Status [N (%)] |  |  |  |  |
| Current Smoker | 5 (4%) | 6 (9.2%) | 4 (2.9%) | 1 (1.3%) |
| Does not smoke | 109 (85.8%) | 51 (78.5%) | 130 (92.9%) | 70 (88.6%) |
| Ex-Smoker | 13 (10.2%) | 8 (12.3%) | 6 (4.3%) | 8 (10.1%) |
| Ethnicity [N (%)] |  |  |  |  |
| White | 121 (95.3%) | 59 (92.2%) | 136 (97.1%) | 77 (98.7%) |
| Non-White | 6 (4.7%) | 5 (7.8%) | 4 (2.9%) | 1 (1.3%) |
| Handedness [N (%)] |  |  |  |  |
| Right | 102 (80.3%) | 47 (77.0%) | 117 (83.6%) | 73 (92.4%) |
| Left | 10 (7.9%) | 7 (11.5%) | 15 (10.7%) | 4 (5.1%) |
| Both | 15 (11.8%) | 7 (11.5%) | 8 (5.7%) | 2 (2.5%) |
| Playing Position [N (%)] |  |  |  |  |
| Wicketkeepers | 17 (13.4%) | 13 (21.3%) | -- | -- |
| Batsmen | 35 (27.6%) | 2 (3.3%) | -- | -- |
| Bowlers/All-rounders | 75 (59.0%) | 46 (75.4%) | -- | -- |
| Fronts | -- | -- | 63 (45.0%) | 39 (53.4%) |
| Backs | -- | -- | 77 (55.0%) | 34 (46.6%) |
| Time Since Retirement (years) |  |  |  |  |
| Mean (SD) | 23.7 (13.7)*† | 27.8 (12.7)† | 27.7 (16.0)* | 29.9 (15.5) |
| Range | 2 - 57 | 6 - 57 | 1 - 63 | 1 - 57 |
| Previous Severe Hand Injury [N (%)] | 46 (36.2%) | 15 (30.0%) | 44 (31.4%) | 22 (26.5%) |

*Denotes differences in characteristic between cricket and rugby samples (p<0.05)

†Denotes differences in characteristic between each sport’s complete case and excluded samples (p<0.05)

**
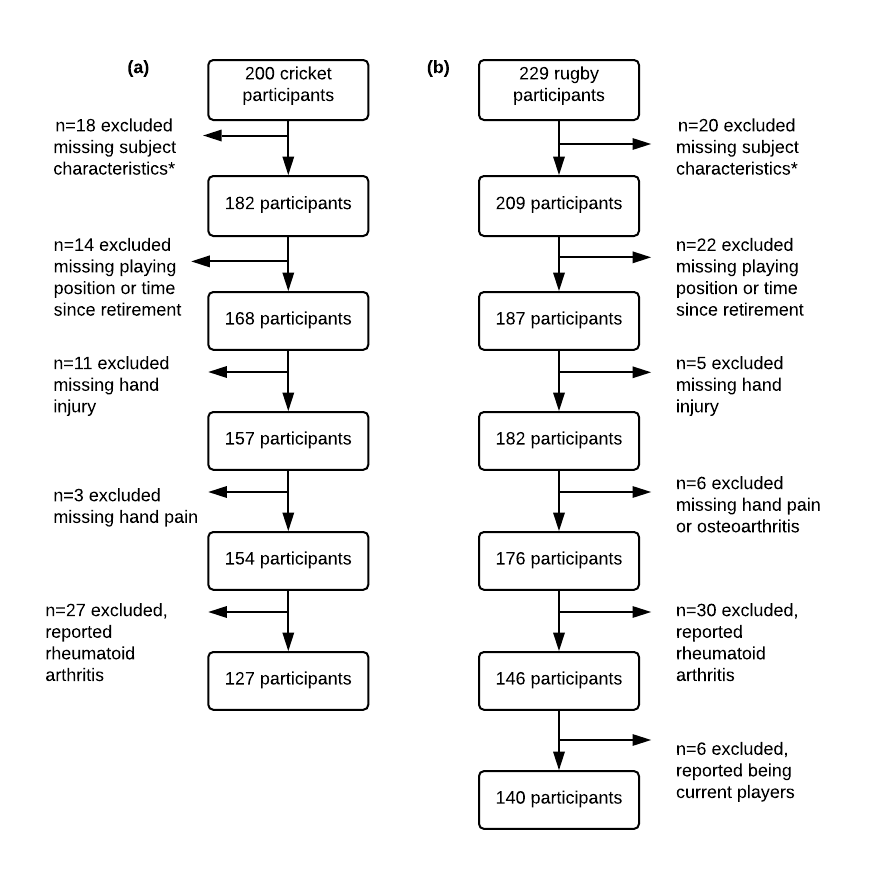
**

*Subject characteristics of age, height, weight, smoking status, ethnicity

**Figure A.1.** Flow chart of former (a) cricket and (b) rugby players excluded from analysis due to missing data or exclusion criteria.
